# Pyrosequencing Reveals the Influence of Organic and Conventional Farming Systems on Bacterial Communities

**DOI:** 10.1371/journal.pone.0051897

**Published:** 2012-12-19

**Authors:** Ru Li, Ehsan Khafipour, Denis O. Krause, Martin H. Entz, Teresa R. de Kievit, W. G. Dilantha Fernando

**Affiliations:** 1 Department of Plant Science, University of Manitoba, Winnipeg, Manitoba Canada; 2 Department of Animal Science, University of Manitoba, Winnipeg, Manitoba, Canada; 3 Department of Medical Microbiology and Infectious Diseases, Winnipeg, Manitoba, Canada; 4 Department of Microbiology, University of Manitoba, Winnipeg, Manitoba, Canada; University of Hyderabad, India

## Abstract

It has been debated how different farming systems influence the composition of soil bacterial communities, which are crucial for maintaining soil health. In this research, we applied high-throughput pyrosequencing of V1 to V3 regions of bacterial 16S rRNA genes to gain further insight into how organic and conventional farming systems and crop rotation influence bulk soil bacterial communities. A 2×2 factorial experiment consisted of two agriculture management systems (organic versus conventional) and two crop rotations (flax-oat-fababean-wheat versus flax-alfalfa-alfalfa-wheat) was conducted at the Glenlea Long-Term Crop Rotation and Management Station, which is Canada’s oldest organic-conventional management study field. Results revealed that there is a significant difference in the composition of bacterial genera between organic and conventional management systems but crop rotation was not a discriminator factor. Organic farming was associated with higher relative abundance of *Proteobacteria*, while *Actinobacteria* and *Chloroflexi* were more abundant in conventional farming. The dominant genera including *Blastococcus, Microlunatus, Pseudonocardia, Solirubrobacter, Brevundimonas, Pseudomonas*, and *Stenotrophomonas* exhibited significant variation between the organic and conventional farming systems. The relative abundance of bacterial communities at the phylum and class level was correlated to soil pH rather than other edaphic properties. In addition, it was found that *Proteobacteria* and *Actinobacteria* were more sensitive to pH variation.

## Introduction

It has long been recognized that maintaining biodiversity of soil microbes is crucial to soil health, which has been defined as soil with the capacity of resilience to stress, sustaining high biological diversity, productivity and high level of internal nutrient cycling, maintaining environmental quality and promoting plant health [1], [2]. Bacterial communities are responsible for multifaceted biological functions in soils [3], [4], [5], and exert an important role in maintaining plant health [6], [7], [8], [9], [10]. In turn, soil has a direct impact on the structure and function of soil bacterial communities through perturbations caused by natural or human activities [11], [12], [13]. It was reported that agricultural soil, perturbed by human activities, has different bacterial diversity, compared to non-disturbed forest and grassland soil [14], [15]. However, there is still lack of the detailed information about the bacterial diversity affected by agriculture perturbation.

Over the past decades, conventional agricultural management practices have involved the use of artificial chemical fertilizers and pesticides to increase crop yields. This has led to severe environmental problems such as soil degradation, emission and leaching of fertilizer and pesticide, and the emergence of pesticide resistant species [16], [17], resulting in an unsustainable practice [18]. The aim in sustainable management systems is to maintain the biological function of the soil and to promote plant health. Organic farming contributes to these factors using techniques such as crop rotation, green manure, and biological pest control instead of chemical fertilizers and pesticides [19]. Consequently, organic farming systems may have a strong potential for restoring soil health and increase agro-ecosystem resilience to stress [18].

Few studies have evaluated the impact of fertilizer, crop rotation, and crop varieties on microbial community structure when conventional and organic farming systems are compared [20], [21], [22], [23], [24]. These studies have found that fertilizers, and crop varieties and rotation could shape the size and structure of soil microbial communities. However, these studies were based on field experiments where the above-mentioned factors varied at the same time between conventional and organic soil management. Therefore, the main discriminator between conventional and organic farming could not be defined. Moreover, the results of these studies were not consistent, which could be due to different analytical methodologies that varied in resolution [14], [24], [25], [26].

Previous studies in our group had investigated the effects of different agriculture management practices (organic versus converntional) and crop rotation systems [(flax-oat-fababean-wheat (Grain-Only rotation) versus flax-alfalfa-alfalfa-wheat (Forage-Grain rotation)] on crop yield, soil edaphic traits, such as nitrate, phosphorus, pH, and organic matter. Results showed that rotation rather than farming managements affect most soil nutrient traits including nitrate-N, Oslen P, and organic matter. Whereas, farming system affect soil pH, with lower pH in conventional farming system than that in organic farming system. However, the effects of farming management and rotation on bacterial communitiy structure of the soil was not evaluated [27], [46].

We hypothesized that microbial composition of soil differs between organic and conventional farming sytstems or in different crop rotations. As such, some microorganisms might be present in organic farms while absent or less frequent in conventional farms and vice versa. Similarly, there might be some bacteria that are unique to a specific crop rotation system. Using Roche 454 pyrosequencing methodology, the objective of this study was to identify bacterial populations that are associated with specific farming practice that could potentially influence soil and plant health. This manuscript provides a detailed framework of the soil bacterial composition at the genus level and its possible connection to farming practices.

## Materials and Methods

### Soil Sampling, Sampling Site and Experimental Design

Soil samples were collected at Glenlea Long-term Crop Rotation and Management Station (GLCRMS) at southern Manitoba, which is Canada’s longest running organic-conventional management comparison station commenced in 1992. A detailed description of the location and site management was described previously [27]. In brief, the study site is located 20 km south of Winnipeg, Manitoba, Canada (N 49,39,0/W 97,7,0). The soil type is Rego Black Chernozem and the soil texture is clay (9% sand, 26% silt, and 66% clay) with an organic matter content of 7.7%. The experiment was a randomized complete block design in a split-plot arrangement with three replicates. Two crop rotations, that is, flax-oat-fababean-wheat (Grain-Only rotation) and wheat-alfalfa-alfalfa-flax (Forage-Grain rotation) were used as main plots, and certificated organic and conventional methods served as subplots. The 2×2 combinations of treatments included: Grain-Only Organic (GO), Grain-Only Conventional (GC), Forage-Grain Organic (FO) and Forage-Grain Conventional (FC). All rotation crops appeared in the rotation each year. Both organic and conventional experiments were managed using conventional tillage and plots were tilled with a disc and a field cultivator prior to sowing. Pesticides and chemical fertilizers were applied on the conventional plots but not on the organic plots. Eighteen kg P_2_O_5_ was banded when wheat was seeded in conventional plots, and 65 kg Nitrogen/ha was broadcasted in conventional flax plots. One L/ha Buctril M and 0.235 L/ha Horizon were sprayed on conventional wheat plots and 0.2 L/ha Select and 1 L/ha Buctril were sprayed on conventional flax plots. These plots were managed with no external input of manure.

Bulk soil samples were randomly collected from the top level (0–15 cm) throughout wheat and flax plots in June and August 2008. Part of each soil sample was kept at −20°C prior to DNA extraction after sieving (2 mm) to remove roots and stones, while the rest was kept at 4°C for chemical analyses. Samples were analyzed using an elemental analyzer at a commercial soil analysis laboratory (AGVISE, Northwood, ND) for total soil carbon and total soil nitrogen (Vario MAX Carbon-Nitrogen analyzer, Elemetar, Germany). Soil Carbonate carbon was analyzed with a modified pressure technique [28]. Organic matter, soil pH, and Olsen phosphorus (P_olsen_): sodium bicarbonate-extractable phosphorus was measured as described by Welsh et al. [27].

### DNA Extraction

To remove PCR inhibitors, such as humic acids, covalent cations and other easily dissolved organic compounds, from soil samples a pre-lysis washing procedure was introduced before DNA extraction [29]. Soil samples of 0.25 g were mixed with 1.25 ml sodium phosphate (0.1 M, pH 7.5), then incubated in a shaker for 1 hr at room temperature, followed by centrifuging for 10 min at 16000×*g*. Supernatant was discarded. DNA was extracted from pre-washed samples using the PowerSoil DNA Isolate kit, which included a bead-beating step, according to the manufacturer’s specifications (Mobio Laboratories, Solana Beach, CA). The DNA purity and quantity were tested by using spectrophotometer (Du 800 Spectrophotometer, BECKMAN COULTER). The average ratio of 260∶280 was 1.7. The average DNA yield was 10 ng/µL. The variable regions of V1–V2 of the 16S rRNA genes were successfully amplified using forward primer 27F (AGAGTTTGATCMTGGCTCAG) and reverse primer 342R (CTGCTGCSYCCCGTAG), indicating that the quality of extracted DNA was sufficient for further PCR application [30]. In order to test the long-term effect of farming practices on the bacterial communities in the soil and reduce the temporal effects of different sampling times, DNA samples of the same treatment collected at different sampling times were pooled before pyrosequencing.

### Pyrosequencing

A total of 23 pooled DNA samples were pyrosequenced using the bacterial tag-encoded GS FLX-Titanium amplicon as described by Dowd et al. [31] and Khafipour et al. [32]. In brief, a mixture of Hot Start, HotStar high fidelity Taq polymerases, and Titanium reagents were used to perform a one-step PCR (35 cycles) with primers 28F (GAGTTTGATCMTGGCTCAG) and 519R (GTNTTACNGCGGCKGCTG), which covered the variable regions V1–V3 of the bacterial 16S rRNA genes [31]. The pyrosequencing procedures were carried out at the Research and Testing Laboratory (Lubbock, TX; http://www.Researchandtesting.com).

### Bioinformatics of Pyrosequencing Data

#### Sequence editing, categorical transformation/classification

Pyrosequencing data were edited, categorically transformed and classified as described by Khafipour et al. [32]. Briefly, all low quality sequences, tags, non-bacterial ribosomal sequences, and chimeras were removed from the database. In total, 123, 316 sequences were generated in this step. Then, the mothur software package [33] was utilized to perform the second round of sequence quality control and assignments of operational taxonomic units (OTU). All sequences shorter than 200 bp, or sequences having one or more ambiguous base, or containing a homopolymer length equal or greater than 8bp were removed from the dataset. The minimum, median and maximum lengths of sequences were 200, 471 and 647 bp, respectively. The unique sequences were then identified and aligned against a database of high quality 16S rRNA bacterial sequences derived from Silva (version 106) [34]. Through screening, filtering, and pre-clustering processes, columns containing a gap were removed in all sequences to reduce noise from pyrosequencing data. The remaining 987 columns (with an actual sequence length varying from 203 to 342 bp) and 39,283 sequences were used to build a distance matrix with a distance threshold of 0.1. Using the furthest neighbor algorithm with a cutoff of 95% similarity, these sequences were clustered to OTU. Representative sequences from each OTU were taxonomically classified with a confidence level of 60% using RDP Bayesian approach [35].

#### Alfa diversity analysis

An OTU-based approach was performed to calculate the richness, diversity and coverage at OTU cutoff of 0.05, which characterizes the biodiversity of the bacterial population in the soil samples at the genus level. Richness indices, Chao1 and abundance based coverage estimation (ACE), were calculated to estimate the number of species or OTU that were present in the sampling assemblage. The diversity within each individual sample, which is made up of richness and species abundance, was estimated using Simpson and non-parametric Shannon diversity indices. Good’s non-parametric coverage estimator was used to estimate the percentage of the total species that were sequenced in each sample. Rarefaction curves for treatment groups were created in mothur [33], based on a re-sampling without replacement approach.

### Statistical Hypothesis Testing

The UNIVARIATE procedure of SAS [36] was used to test the normality of residuals for Alfa diversity indices. Non-normally distributed data were power transformed using Box-Cox power transformation macro (http://www.datavis.ca/sasmac/boxcox.html) in SAS based on the following models: BoxCox (y) = (y^λ^ - 1)/λ, if λ≠0 OR BoxCox (y) = log (y), if λ = 0. The range of λ for each parameter was adjusted by trial and error and the best fitting value of λ was identified using maximum likelihood methods. Normalized data were then used to assess the effect of treatment using MIXED procedure of SAS [36]. The effect of replicates was treated as random in the model.

Percentage data was used to evaluate statistical differences among treatments at the phylum and genus levels. To do so, the count data for each taxon was first transformed to the percentage of that taxon in an individual sample. Then UNIVARIATE procedure of SAS was used to test the normality of residuals for percentage data at each taxonomic level. For non-normally distributed data, Poisson and negative binomial distributions were fitted in the GLIMMIX procedure of SAS [36] to assess the effect of treatment. A log link function was specified for Poisson and negative binomial distributions. The goodness of fit for different distributions was compared using Pearson chi-square/DF (closer to 1 is better). Taxa were categorized as abundant and low-abundant in order to characterize them within each treatment. All taxa above 1% of the population were considered abundant and those below 1% were classified as less-abundant [32]. The differences between treatments were considered significant at *P*<0.05 while trends were observed at *P*<0.1.

### Partial Least Square Discriminant Analysis and Redundancy Analysis

Partial least square discriminant analysis (PLS-DA; SIMCA-P+12.0.1, Umetrics, Umea, Sweden) [37] was performed on genus data to identify the effects of crop rotation and management on the bacterial community. The PLS-DA is a particular case of partial least square regression analysis in which Y is a set of binary (0 versus 1) variables describing the categories of a categorical variable on X. In this case, X variables were bacterial genera and binary Y was observations of organic (1) versus conventional (0), or Grain-Only (1) versus Forage-Grain (0) treatments. For this analysis, data were scaled using Unit Variance in SIMCA-P+ [37]. Cross-validation was then performed to determine the number of significant PLS components and a permutation testing was conducted to validate the model. To avoid over parameterization of the model, variable influence on projection value (VIP) was estimated for each genus and genera with VIP<0.35 were removed from the final model [38], [39]. R^2^X and R^2^Y estimates were then used to evaluate the goodness of fit and Q^2^ estimate was used to evaluate the predictive value of the model. Scatter- and score-plots were generated only for treatments that were significantly differentiated by the model. The PLS regression coefficients were used to identify genera that were most characteristic of each treatment group. The positive or negative correlations were considered significant when there was no overlap between the genus 95% confidence interval and the horizontal axis in the PLS regression coefficients graph.

Redundancy analysis (RDA) was carried out using canonical community ordination (CANOCO; Plant Research International BV, Wageningen, The Netherlands) to examine the relationship between abundant phyla and environment variables. Spearman’s rank correlations were used to correlate abundant phyla and soil properties using SAS [36].

## Results

### Cropping Systems and Edaphic Soil Properties

The total soil C, N, and C: N ratio did not vary significantly under different cropping systems, while pH, organic matter, carbonate C and Olsen P were affected to varying degree by cropping systems. Total soil C was 3.0 g/kg, 3.2 g/kg 3.1 g/kg and 3.0 g/kg under GO, GC, FO, and FC farming systems, respectively. Total soil N was 2.7 g/kg in all of four treatments. C: N ratio was 11.3 under GO and FO farming systems, while it was 11.5 and 10.9 under GC and FC farming systems, respectively. In contrast, pH was 7.0 under organic management systems, higher than that of conventional systems with 6.7 (*P* = 0.023). Organic matter was 7.9 under Forage-Grain rotation systems, significantly higher than that of Grain-only rotation systems with 6.7 and 7.2 (*P* = 0.005). Both rotation and management affected carbonate C and Olsen P (Table 1). Carbonate C and Olsen P were much lower under Forage-grain rotation, compared to Grain-only system. Significant correlations existed between total N, C, carbonate C, organic matter and Olsen P, as well as between organic matter, carbonate C, total C and total N (Table 2).

**Table 1:** Physico-chemical characteristics of Glenlea soil (0–15 cm) on the different treatments (Welsh, 2009; Bell 2012).

Rotation	Management	Total C(g/kg)	Carbonate C (g/kg)	Organic matter (%)	Total N(g/kg)	Olsen P (mg/kg)	pH	C: NRatio
Grain-Only	Organic	3.0	2.8	6.7	2.6	23.4	7.0	11.3
Grain-Only	Conventional	3.2	2.6	7.2	2.7	21.7	6.6	11.5
Forage-Grain	Organic	3.1	0.4	7.9	2.7	3.6	7.0	11.3
Forage-Grain	Conventional	3.0	1.7	7.9	2.7	14.2	6.6	10.9
SEM		0.06	0.19	0.50	0.008	1.63	0.32	0.19
*P*-value								
Rotation		NS	<0.0001	0.005	NS	<0.0001	NS	NS
Management		NS	0.0039	NS	NS	0.0035	0.023	NS
Management × Rotation		0.01	0.0001	NS	NS	0.0001	NS	NS

NS =  not significant (*P*>0.05).

**Table 2:** Pearson correlation coefficients between soil edaphic factors^1^.

Variables	pH	Olsen P	Total N	Total C	Carbonate C	Organic matter	C: N ratio
pH	1.000	−0.380	−0.414	−0.429	−0.527	−0.424	−0.205
Olsen P		1.000	**0.996**	**0.993**	**0.615**	**0.994**	0.256
Total N			1.000	**0.998**	**0.606**	**0.998**	0.258
Total C				1.000	**0.622**	**1.000**	0.297
Carbonate C					1.000	**0.611**	0.135
Organic matter						1.000	0.297
C: N ratio							1.000

1 Significant correlations between edaphic factors are indicated in bold type when *P*<0.05.

### Bacterial α-diversity

Bacterial diversity and richness in individual samples under different treatments were calculated (Table 3). Statistical differences in richness and diversity were only observed for coverage and ACE at the management level. Percentage of coverage for conventional treatment was higher than that of organic treatment (*P* = 0.04). The GC had the highest percentage of coverage (84.5%), followed by FC (80.3%), GO (78.8%), and FO (73.2%). The ACE richness was highest for FO (6,147.9), and lowest for GC (3,044.2). The rarefaction curve (Figure S1) generated with mothur demonstrated that observed numbers of OTU of FO and GO groups were higher than that of FC and GC groups, with FO having the highest number of observed OTU.

**Table 3:** Summary statistics of pyrosequencing 16S rRNA sequences of soil samples.

Rotation	Management	Number of trimmed sequences	Mean (SEM) results for indicated variable^1^						
			OTU^2^ (95% distance)	Coverage (%)	Richness^3^		Diversity^4^		
					Chao1	ACE	Shannon	Simpson	Effective number of species
Grain-Only	Organic	30,482	1,917	78.8^a,b^	3,660.0	4,936.4^a,b^	7.2	0.0012	562.8
	Conventional	20,473	1,628	84.5^a^	2,889.7	3,044.2^b^	6.8	0.0022	504.4
Forage-Grain	Organic	23,923	2,118	73.2^b^	4,308.7	6,147.9^a^	7.6	0.0005	681.3
	Conventional	31,552	1,860	80.3^a,b^	3,494.4	4,533.6^a,b^	7.2	0.0012	592.2
SEM			236.5	2.7	512.1	677.1	0.22	0.01	122.1
*P*-value									
Rotation			0.35	0.11	0.20	0.08	0.20	0.38	0.43
Management			0.30	0.04	0.13	0.03	0.20	0.41	0.57
Rotation × Management(*P-*value)			0.95	0.83	0.96	0.83	0.97	0.86	0.91

a, b, c Means with different letters are significantly different for management at *P*<0.05.

1 Mean are from statistical models based on 5 to 6 replicate samples.

2 OTU =  operational taxonomic units.

3 Based on Chao1 and abundance based coverage estimation (ACE) richness indices.

4 Based on Shannon and Simpson diversity estimators.

### Bacterial Community Composition

A total of 14 bacterial phyla were found in all the samples, of which seven were abundant (>1%) (Table 4). Ninety six percent of soil bacterial sequences belonged to these abundant phyla including*: Proteobacteria*, *Actinobacteria*, *Acidobacteria*, *Gemmatinomadetes*, *Chloroflexi*, *Bacteroidetes* and *Planctomycetes*. *Firmicutes*, *Fibrobacteres*, *Nitrospirae*, *Verrucomicrobia*, *P10*, *TM7* and *WS3* were in low abundance. The phylum distribution fluctuated under different farming disturbances. *Proteobacteria* accounted for 44.5% of total bacterial communities under the GO system, while it was only 27.3% under FC. In contrast, *Actinobacteria* made up 43.1% of total bacterial communities under the FC system, but were present in lower percentage (32.5%) under GO. Phylum *Chloroflexi* was also significantly influenced by management, with the highest percentage (6.8%) found in FC compared to the lowest (3.5%) in GO. A significant interaction between rotation and system was observed for *Gemmatinomadetes*, *Fibrobacteres*, *Verrucomicrobia*, and *P10*. Percentage of *Nitrospirae* was higher under Forage-Grain farming system. Other phyla did not show significant differences under different treatments. Unassigned bacterial sequences at the phylum level were approximately 1% of the total. In total, eight out of 14 phyla showed significant differences under different farming systems.

**Table 4:** Phylogenetic composition of bacterial phyla from pyrosequenced 16S rRNA sequences.

Phylum	Rotation (Grain-Only)			Rotation (Forage-Grain)		SEM	*P*-value		
	Management						Rotation	Management	Rotation × Management
	Organic	Conventional		Organic	Conventional				
	Abundant phyla^1^								
*Proteobacteria*	44.5^a^	32.2^a,b^		34.1^a,b^	27.3^b^	3.13	0.10	0.05	0.70
*Actinobacteria*	32.5^a,b^	39.2^a,b^		28.4^b^	43.1^a^	2.21	0.97	0.0002	0.08
*Acidobacteria*	8.5	10.3		13.8	12.1	2.35	0.15	0.88	0.44
*Gemmatinomadetes*	3.5^B^	3.6^B^		8.6^A^	2.9^B^	1.16	0.15	0.06	0.04
*Chloroflexi*	3.4^b^	6.1^a^		5.2^a,b^	6.8^a^	0.98	0.18	0.04	0.42
*Bacteroidetes*	3.3	2.7		2.7	2.2	0.98	0.54	0.52	0.99
*Planctomycetes*	1.5	1.7		2.1	1.8	0.35	0.30	0.81	0.49
	Low-abundance phyla^2^								
*Firmicutes*	0.6		0.9	0.2	1.1	0.34	0.47	0.11	0.32
*Fibrobacteres*	0.1^B^		0.2^B^	0.3^A^	0.1^B^	0.04	0.05	0.07	0.01
*Nitrospirae*	0.2^b^		0.2^a,b^	0.3^a,b^	0.5^a^	0.07	0.01	0.21	0.35
*Verrucomicrobia*	0.2^C^		0.7^A^	0.8^A^	0.5^B^	0.14	0.09	0.42	0.006
*OP10*	0.2^B^		0.4^A,B^	0.5^A^	0.2^B^	0.08	0.44	0.36	0.04
*TM7*	0.1		*	0.1	*	0.13	0.98	0.62	0.86
*WS3*	*		0.2	0.2	0.1	0.15	0.59	0.85	0.56
Unclassified	1.1		1.3	2.1	1.3	0.52	0.45	0.66	0.40

a,b,c Means for main effects (rotation or management) are significantly different at *P*<0.05.

A, B, C Means for the interaction between rotation and system are significantly different at *P*<0.05.

1 Percentage of sequences larger than 1.

2 Percentage of sequences smaller than 1.

* Percentage of sequences below 0.1.

The relative abundance of different genera showing significant difference under different treatments was listed in Table 5. In phylum *Actinobacteria*, several putative genera including *Blastococcus*, *Lapillicoccus*, *Microlunatus, Pseudonocardia*, *Solirubrobacter*, and *Rubrobacter* showed significant differences among the treatments. The relative abundance of these genera was highest in the FC farming system, followed by GC, FO, and GO. The percentage of different class and genera belonging to the phylum *Proteobacteria* was higher under organic farming system compared to the conventional farming conditions with the exception of *Skermanella*, which was 2.6% and 1.5% under FC and GC farming systems, respectively. Classes *Alphaproteobacteria*, *Betaproteobacteria* and *Gammaproteobacteria* were higher in the Grain-Only organic farming system, although the difference was not statistically significant. Class *Deltaproteobacteria* showed the opposite pattern, being highest in Forage-Grain conventional systems, and lowest in Grain-Only organic system. *Pseudomonas* was the predominant genus in *Gammaproteobacteria* with 4.3% in GO, 3.9% in GC, 1.7% in FO, and 0.5% in FC. Within Phylum *Chloroflexi*, genus *Roseiflexus* was significantly influenced by interaction of rotation and management. Other genera in the phyla of *Actinobacteria* and *Proteobacteria* did not show statistical variation among the treatments (Table S1 and Table S2). There was no significant fluctuation under different farming systems in other genera within *Acidobacteria*, *Bacteroidetes*, *Firmicutes*, and *Planctomycetes* (Table S3).

**Table 5:** Bacterial taxa showing significant variation under different farming systems generated using pyrosequenced 16S rRNA sequences.

Taxa (family and genus within each phylum or class)	Rotation (Grain-Only)		Rotation (Forage-Grain)				SEM	*P*-value				
	Management							Rotation		Management		Rotation × Management
	Organic	Conventional		Organic		Conventional						
***Actinobacteria***	32.5^a,b^	39.2^a,b^	28.4^b^		43.1^a^		2.21		0.97	0.0002	0.08	
Geodermatophilaceae; *Blastococcus*	0.8^b^	1.7^a^	1.2^a,b^		1.7^a^		0.19		0.27	0.002	0.23	
Intrasporangiaceae; *Lapillicoccus*	0.3^b^	0.4^a,b^	0.2^b^		0.6^a^		0.09		0.36	0.01	0.11	
Propionibacteriaceae; *Microlunatus*	1.1^a.b^	2.0^a^	0.8^b^		2.1^a^		0.31		0.78	0.004	0.66	
Pseudonocardiaceae; *Pseudonocardia*	0.9^b^	1.7^a,b^	1.3^b^		2.6^a^		0.24		0.02	0.001	0.31	
Solirubrobacteriaceae; *Solirubrobacter*	0.6^b^	1.7^a^	0.8^b^		1.6^a^		0.29		0.88	0.003	0.48	
Rubrobacteriaceae; *Rubrobacter*	0.5^b^	1.0^a^	0.3^b^		1.4^a^		0.24		0.57	0.005	0.30	
Unclassified bacteria	11.9^a,b^	15.6^a,b^	9.9^b^		17.4^a^		1.81		0.97	0.01	0.33	
***Proteobacteria***	44.5^a^	32.2^a,b^	34.1^a,b^		27.3^b^		3.13		0.10	0.05	0.70	
*** Alphaproteobacteria***	20.5	15.9	18.2		15.0		2.39		0.55	0.15	0.85	
Caulobacteraceae; *Brevundimonas*	1.7^a^	0.1^b^	0.2^b^		0.01^b^		0.11		0.15	0.03	0.78	
Xanthobacteraceae; uncultured	0.5^b^	0.8^a,b^	0.7^a,b^		1.1^a^		0.15		0.10	0.05	0.64	
Rhodospirillaceae; *Skermanella*	0.8^b^	1.5^a,b^	1.3^ab^		2.6^a^		0.30		0.02	0.005	0.36	
*** Gammaproteobacteria***	11.4	7.1	7.7		3.6		2.72		0.13	0.08	0.67	
Pseudomonadaceae; *Pseudomonas*	4.3	3.9	1.7		0.5		2.02		0.10	0.52	0.44	
Xanthomonadaceae; *Stenotrophomonas*	0.7^a^	0.0^b^	0.3^a^		0.0^b^		0.25		0.67	0.04	0.71	
*** Betaproteobacteria***	10.3^a^	6.3^b^	8.7^a,b^		5.1^b^		2.00		0.42	0.04	0.93	
Burkholderiaceae; *Burkholderia*	0.6	0.0	0.1		0.0		0.19		0.29	0.14	0.99	
*** Deltaproteobacteria***	1.0^b^	2.7^a,b^	3.4^a^		3.5^a^		0.05		0.009	0.10	0.13	
***Chloroflexi***	3.4^b^	6.1^a^	5.2^a,b^		6.8^a^		0.98		0.18	0.04	0.42	
Chloroflexaceae; *Roseiflexus*	0.6	1.5	1.1		2.3		0.28		0.02	0.002	0.52	

a,b,c Means for main effects (rotation or management) are significantly different at *P*<0.05.

A, B, C Means for the interaction between rotation and system are significantly different at *P*<0.05.

1 Percentage of sequences larger than 1.

2 Percentage of sequences smaller than 1.

* Percentage of sequences below 0.1.

**Figure 1:**
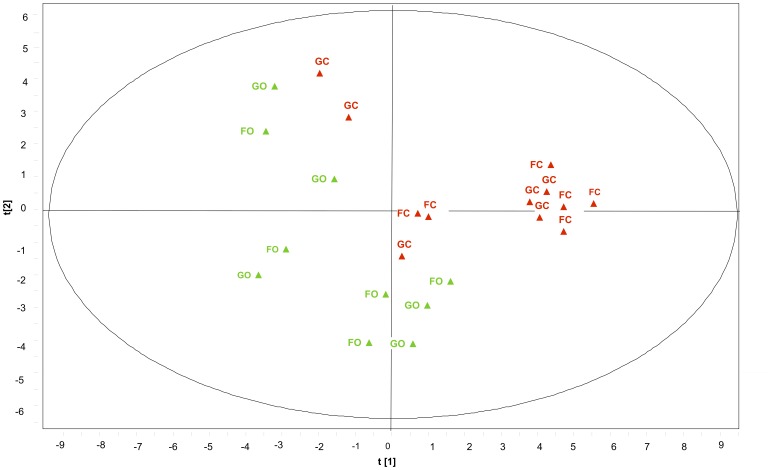
Partial least square discriminant score plot of soil bacteria under organic and conventional treatments. GO: Grain-Only organic; GC: Grain-Only conventional; FO: Forage-Grain organic; FC: Forage-Grain conventional. Model indicated a significant difference in the composition of putative bacterial genera between organic and conventional managements (R^2^X = 0.427, R^2^Y = 0.882, Q^2^ = 0.159). Only genera with VIP>0.35 is included in the model.

**Figure 2:**
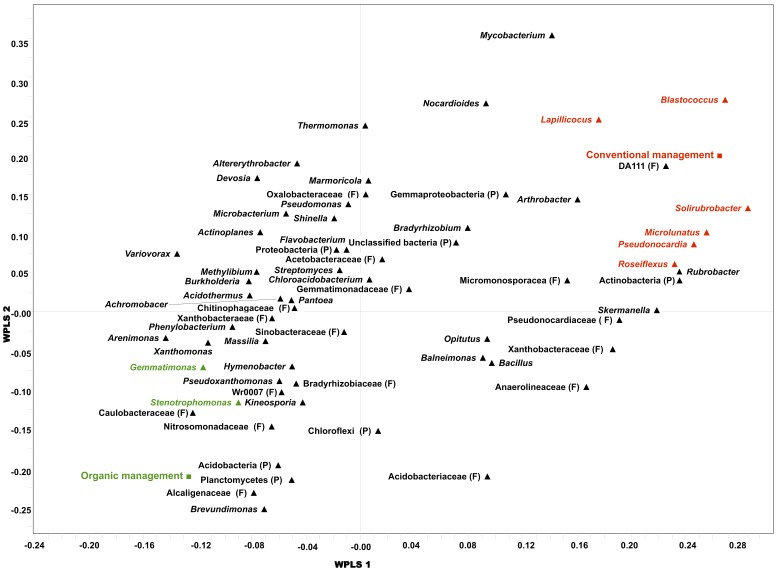
Partial least square discriminant analysis (PLS-DA) loading plot based on the relative abundance of the putative bacterial genera in soil microbiome and their association with organic or conventional treatments. Bacterial genera closer to organic or conventional are highly correlated to either treatment. PLS1 (R^2^X = 0.27, R^2^Y = 0.525, Q^2^ = 0.186) and PLS2 (R^2^X = 0.127, R^2^Y = 0.218, Q^2^ = −0.081). Some sequences could only be affiliated to phylum (P) or family (F) levels.

The PLS-DA analysis showed that there is a significant difference in the composition of bacterial genera between organic and conventional managements (R^2^X = 0.427, R^2^Y = 0.882, Q^2^ = 0.159) (Figure 1). However, crop rotation (Forage-Grain versus Grain-Only) was not a discriminator factor. Genera that were the most characteristic of each management system were identified using a scatter-plot (Figure 2). Among the putative bacterial genera included in the model, *Blastococcus* spp. *Microlunatus* spp. *Pseudonocardia* spp. and *Solirubrobacter* spp. were significantly correlated with conventional treatment, whereas *Gemmatimonas* spp. and *Stenotrophomonas* spp. were highly associated with organic management (Figure 2 and Figure S2).

### Effect of Soil Edaphic Properties on Abundant Phyla

**Figure 3:**
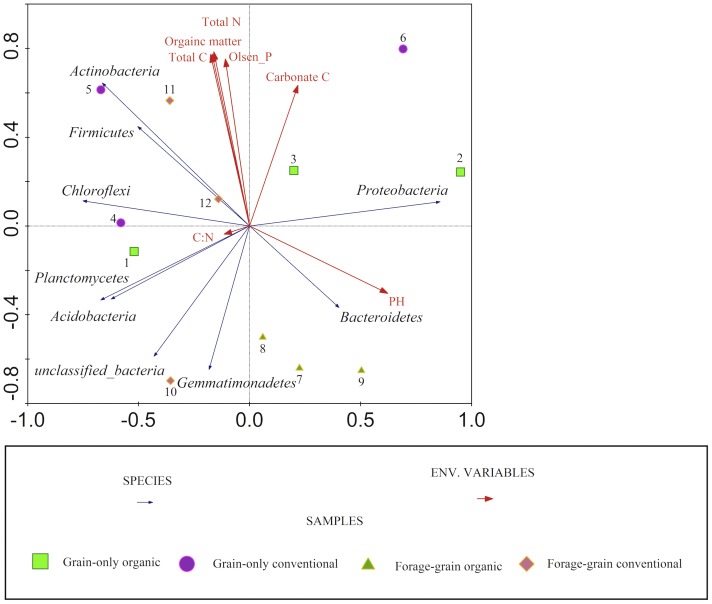
Redundancy analysis ordination plots of abundant phyla for individual sample.

Canonical correspondence analysis tested the effect of soil edaphic properties on samples and bacterial populations by using an unconstrained analysis (RDA) (Figure 3). pH explained 24% of the variance (*P* = 0.06), CaCO3 C explained 19% (*P* = 0.02), and the C: N ratio accounted for less than 5% of the variance (*P* = 0.52). Other soil edaphic variables were highly correlated with each other and were not able to explain variance separately. We also used Spearman’s rank order correlation to evaluate relationships between abundant phyla and soil edaphic properties (Table 6). It was found that the relative abundance of *Proteobacteria* phylum and *Betaproteobacteria* class was positively correlated with soil pH, while the abundance of *Actinobacteria* was negatively correlated with soil pH. Other phyla did not show significant correlations with soil edaphic properties.

**Table 6:** Spearman’s rank correlations between abundant phyla with soil properties^1^.

Abundant phyla	Correlation						
	pH	Olsen P	Total N	Total C	Carbonate C	Organic matter	C: N ratio
*Proteobacteria*	**0.61**	0.07	−0.04	−0.03	−0.06	−0.03	0.05
*Alphaproteobacteria*	0.46	−0.47	−0.09	−0.12	−0.17	−0.11	−0.17
*Betaproteobacteria*	**0.62**	−0.18	−0.24	−0.38	−0.36	−0.20	−0.36
*Gammaproteobacteria*	0.25	−0.004	−0.05	−0.15	−0.13	0.04	−0.13
*Deltaproteobacteria*	−0.47	0.18	−0.21	−0.15	−0.13	−0.04	−0.13
*Actinobacteria*	−**0.65**	0.44	0.51	0.50	0.41	0.50	−0.17
*Bacteroidetes*	0.33	−0.37	−0.37	−0.36	−0.53	−0.36	0.38
*Chloroflexi*	−0.53	0.33	0.40	0.39	0.40	0.39	0.09
*Firmicutes*	−0.21	0.47	0.53	0.52	0.33	0.52	0.35
*Gemmatimonadetes*	−0.08	−0.46	−0.45	−0.45	−0.30	−0.45	−0.03
*Planctomycetes*	−0.18	−0.28	−0.21	−0.24	−0.31	−0.24	−0.16
*Acidobacteria*	−0.06	−0.23	−0.16	−0.20	−0.35	−0.20	−0.15
*Nitrospirae*	−0.004	−0.24	−0.19	−0.27	−0.40	−0.27	−0.23
Unclassified	−0.39	−0.47	−0.41	−0.41	−0.19	−0.41	−0.14

1 Significant correlations between edaphic factors are indicated in bold type when *P*<0.05.

## Discussion

In this study, crop rotation and management strategies did not alter total C, total N and C:N ratio but significantly affected organic matter, soil pH, carbonate C and Olsen P (Table 1). This indicates that farming systems gradually but not dramatically change soil edaphic properties [40]. We observed that organic management led to a neutral soil pH compared to conventional practices (7.0 versus 6.6; Table 1). This might be due to the application of synthetic fertilizer that could acidify the soil in the conventional systems [41]. Other reports indicated that soil pH could be influenced by other soil traits such as C:N ratio [42], vegetation, or soil type [43]. However, in our study, the field trials were run under identical condition, and the soil pH was not significantly correlated with other soil edaphic characteristics (Table 2). Therefore, the farming system was the sole factor to change the soil pH. As we expected, soil organic matter was higher under Forage-Grain rotations (Table 1) [42], [44]. However, organic farming system did not increase organic matter in the soil surface, compared to conventional farming system. In contrast, other studies have shown that organic matter was higher in the top 0.3 m of soil under organic management [21], [45]. This discrepancy could be due to different crops contributing to different amount of biomass and no additional manure added to our trials [46].

We used high-resolution power of 454-pyrosequencing to obtain insight into the effects of farming management styles (organic, conventional) and crop rotations (Grain-Only, Forage-Grain) on the diversity, richness and composition of soil bacterial communities. In total, pyrosequencing identified 14 phyla and 178 putative genera of bacteria in different soil samples. We found that organic and conventional farming management had major influence on soil bacterial communities while the effects of crop rotation were of smaller magnitude. We were also able to identify putative genera that were correlated with either organic or conventional farming management.

It has been reported that organic farming systems enhance microbial diversity in soil compared to the conventional systems [21]. Although not statistically significant, we found a similar trend in this study (Table 3). Previous research indicated that soil pH might be the primary factor influencing richness and diversity of bacterial communities [47] with the highest richness and diversity found to be near the neutral pH. Lauber et al. [48] proposed that bacterial diversity had a strong negative relationship with soil pH when it was lower than 6.5. In this study, soil pH ranged from 6.6 to 7.0, and was significantly higher for organic compared to conventional system. This indirectly indicates that organic management might favor higher bacterial diversity. And it is important to notice that standard diversity parameters only based on OTU without taxonomic identity of the different groups is not sensitive enough to detect the influence of agriculture management on the soil bacterial community, because changes in some taxonomic groups might be compensated by changes in others [49]. Lending support to this hypothesis, we detected a significant shift in soil bacterial communities due to farming systems when sequences were taxonomically ranked (Figure 1).

When sequences were affiliated to taxonomic level, bacterial populations fluctuated under different farming systems. An interesting observation in this study was the greater percentage of phylum *Proteobacteria*, including classes *Alphaproteobacteria*, *Betaproteobacteria* and *Gammaproteobacteria* in organic farming management (39.3%) compared to the conventional system (29.7%). To interpret these findings in an ecological context and to explain why some bacterial phyla are more abundant in soil than others, some researchers have used the concept of copiotrophic versus oligotrophic bacteria [50], [51]. Copiotrophic bacteria (fast growing) flourish in soils with large amounts of available nutrients, while oligotrophic groups (slow growing) predominate in soil having low nutrient availability. It has been proposed that oligotrophic bacteria are more associated with organically than conventionally farmed soils due to low availability of organic carbon and nitrogen [2], [40]. Among *Proteobacteria*, *Betaproteobacteria* are considered as copiotrophic [40], [50], and thus, their population is expected to be lower in organic farming. There is no indication if other classes within *Proteobacteria* can be classified into copiotrophic-oligotrophic scheme [50]. In our study we found higher *Betaproteobacteria* in organic farmed soil. As organic farming system did not contribute to higher amount of top bulk soil total C, total N and organic matter compared to conventional system, higher relative abundance of these bacteria could be due to other factors. It was found that *Proteobacteria* and *Betaproteobacteria* was highly correlated with pH in this study (P<0.05, Table 6), we assumed that neutral pH could increase the abundance of these bacteria in soil.

We believe that because of enormous phylogenetic and physiological diversity within each bacterial phyla, it is unlikely that an entire phylum demonstrate same ecological characteristics. An example would be *Burkholderia*, a genus in *Betaproteobacteria* that exhibits oligotrophic traits due to their catabolically versatility that enables them to degrade recalcitrant compounds and survive in environments with limited nutrient availability [52]. Thus, the hypothesis that oligotrophic bacteria are more associated with organic farmed soil could simplify the ecological categories of bacterial communities in soil.

In this study, we found a higher population of *Brevundimonas* spp., *Burkholderia* spp., *Pseudomonas* spp., and *Stenotrophomonas* spp. in organic farming systems (Table 5). These genera are ubiquitously in the soil and several of their species have important ecological roles in nutrient cycling and suppression of plant diseases [52], [53], [54]. For instance, members of *Stenotophomonas, Pseudomonas and Burkholderia* genera can fix nitrogen [52], [55]. Higher relative abundance of these genera might help maintaining total N level in organic farming soil without fertilizer supplementation. In addition, many plant growth-promoting bacteria (PGPB) belong to *Burkholeria*, *Stenotrophomonas* and *Pseudomonas* genera, which were more abundant in organic farming system (Table 5). Interestingly, these genera were abundant in soils planted with alfalfa, wheat, oilseed rape and various weeds [53], [54]. Because organic farming systems support more weeds than the conventional farming systems, it might promote these PGPB populations [56]. However, it is important to notice that not all species in these genera are PGPB and there are species, which are pathogenic to humans, animals and plants (i.e. *P. aerugionsa*, *P. syringae*, and *S. maltophilia* K279a*)*. The 16S rRNA marker genes have limitation for identification of bacteria up to the species level, and thus other methodologies with high resolution including metagenomic shotgun sequencing [57] must be applied in order to differentiate PGPB from pathogenic species in the soil bacterial community.

The percentage of *Actinobacteria* and *Chloroflexi* were lower in organic (30.4% and 4.3%, respectively) compared to the conventional system (41.1% and 6.4%, respectively). Our results show that the conventional farming system increases the actinobacterial proportion in the community with no change in their composition, compared to the organic farming system. The PLS-DA loading scatter and coefficient plots (Figure 2 and Figure S2) indicated that conventional farming system supported higher population of several genera within *Actinobacteria*, including *Blastococcus* spp., *Microlunatus* spp., *Pseudonocardia* spp. and *Solirubrobacter* spp. *Actinobacteria* are able to degrade a variety of organic compounds including some herbicides and pesticides [58]. *Pseudonocardia* spp. has been reported to degrade environmental contaminants, particularly aromatic hydrocarbons or compounds that contain aromatic rings [59]. As such, herbicides and pesticides sprayed containing aromatic rings may have favored bacteria, such as *Pseudonocardia* spp., with specific metabolic capabilities that can degrade them. Some *Microlunatus* spp. has high levels of phosphorus accumulating function and phosphate uptake/release activities [60]. Therefore, in a conventional farming system where pesticides and inorganic fertilizers are commonly used to increase the crop yield, high availability of substrate for *Microlunatus* spp. and other actinobacterial species could boost their population.

*Actinobacteria* also play a major role in organic matter turnover and carbon cycling. They can decompose some recalcitrant carbon sources including cellulose and chitin [15], [61]. Organically farmed soils have been reported to be rich in recalcitrant carbon sources [62], and the diversity of *Actinobacteria* would be expected to be higher in those soils than in conventionally farmed soils. However, in our organic farming fields the recalcitrant carbon sources were not higher in the organically surface soil than the conventional one [46]. Therefore, recalcitrant carbon sources could not drive the increasing diversity of *Actinobacteria* in our study.

A number of studies have shown that soil edaphic factors shaped microbial communities [43], [63], [64], [65]. In our study, we found that proportions of abundant phyla were highly affected by soil pH. Our observations were consistent with other studies that demonstrated pH was one of the main drivers of change in soil bacterial communities from continental scale [48] to small landscape [64], [65]. At the phylum level, *Proteobacteria* were positively correlated with soil pH, while *Actinobacteria* were negatively correlated and *Acidobacteria* had a very weak correlation with soil pH. Our results are in contrast to some studies that showed *Actinobacteria* significantly increased with higher pH values, and *Acidobacteria* was dependent on soil pH [65], [66]. The soil pH value varied significantly from 3 to 8 in other studies, while the soil pH in our experiments only varied from 6.6 to 7 which could be the reason for lack of change in *Acidobacteria* populations. In our study, *Betaproteobacteria* and *Alphaproteobacteria* populations increased with higher soil pH, while *Deltaproteobacteria* declined. This result was concomitant with the study by Nacke et al. [65]. Our studies demonstrated that *Proteobacteria* and *Actinobacteria* were more sensitive to pH variation than other bacterial phyla.

Finally, it is important to acknowledge that the choice of target variable regions of 16S rRNA may have affected the outcome of species richness and diversity analyses because the sequence divergence is not distributed evenly along the 16S rRNA gene [67], [68]. We deep sequenced the V1–V3 regions of the bacterial 16S rRNA, which covered V2–V3 region, most suitable for distinguishing most bacterial species ranging from the phylum level to the genus level [68], [69]. Therefore, even if some bacterial communities might have been missed or overestimated, the overall shifting in the phylogenetic composition of bacterial communities under different treatments have been assessed.

### Conclusion

We demonstrated that different farming practices significantly changed the relative abundances of *Proteobacteria* and *Actinobacteria*. Farming management practices (organic versus conventional) rather than crop rotation (Grain-Only versus Forage-Grain) appeared to have a strong impact on shifting the abundance of soil bacterial communities, which could translate to changes in soil quality and productivity. Some bacterial groups, such as *Gemmatinomadetes*, *Fibrobacteres*, *Verrucomicrobia* and *OP10* were influenced by the interaction of crop rotation and management. Most soil properties including C: N ratio, total N, total C, Olsen P, and organic matter, did not play a major role in shaping bacterial communities. However, pH had the strongest effect on the bacterial community structure. Organic farming systems led to a neutral pH, which might be beneficial to *Proteobacteria*. On the other hand, conventional farming systems supported a higher percentage of *Actinobacteria*. Therefore, neither organic farming nor conventional farming can address all the aspects of beneficial soil bacterial communities, which is crucial to soil quality and productivity. Further research is required to investigate the shifts in diversity of beneficial bacterial and fungal pathogens under different farming systems in the long run.

## Supporting Information

Figure S1 Rarefaction curves for pooled samples within each treatment at OTU cutoff of 0.05 distance.
(TIF)

Figure S2 Coefficient plot of the bacterial profiles of organic and conventional treatments. Partial least squares discriminant analysis (PLS-DA) coefficient plot based on the relative abundant of the bacterial genera in the microbiome profile of organic and conventional treatments. Genera with significantly positive (>0) or negative (<0) PLS regression coefficients (i.e. no overlap between the 95% confidence interval indicated and the horizontal axis) contribute significantly to the prediction of the organic (green bars) or conventional samples (red bars).
(TIF)

Table S1 Phylogenetic composition of putative bacterial genera in *Actinobacteria* phylum determined using 16S rRNA pyrosequencing
(DOC)

Table S2 Phylogenetic composition of putative bacterial genera in *Proteobacteria* phylum determined using 16S rRNA pyrosequencing
(DOC)

Table S3 Phylogenetic composition of putative bacterial genera in *Acidobacteria*, *Bacteroidetes*, *Chloroflexi*, *Firmicutes*, *Gemmatimonadetes*, and *Planctomycetes* phyla determined using 16S rRNA pyrosequencing
(DOC)
