# Supplementary figures and images for: Pyrosequencing Reveals the Influence of Organic and Conventional Farming Systems on Bacterial Communities
Source: PLoS One. 2012 Dec 19;7(12):e51897. doi: 10.1371/journal.pone.0051897 (PMC3526490; doi:10.1371/journal.pone.0051897)

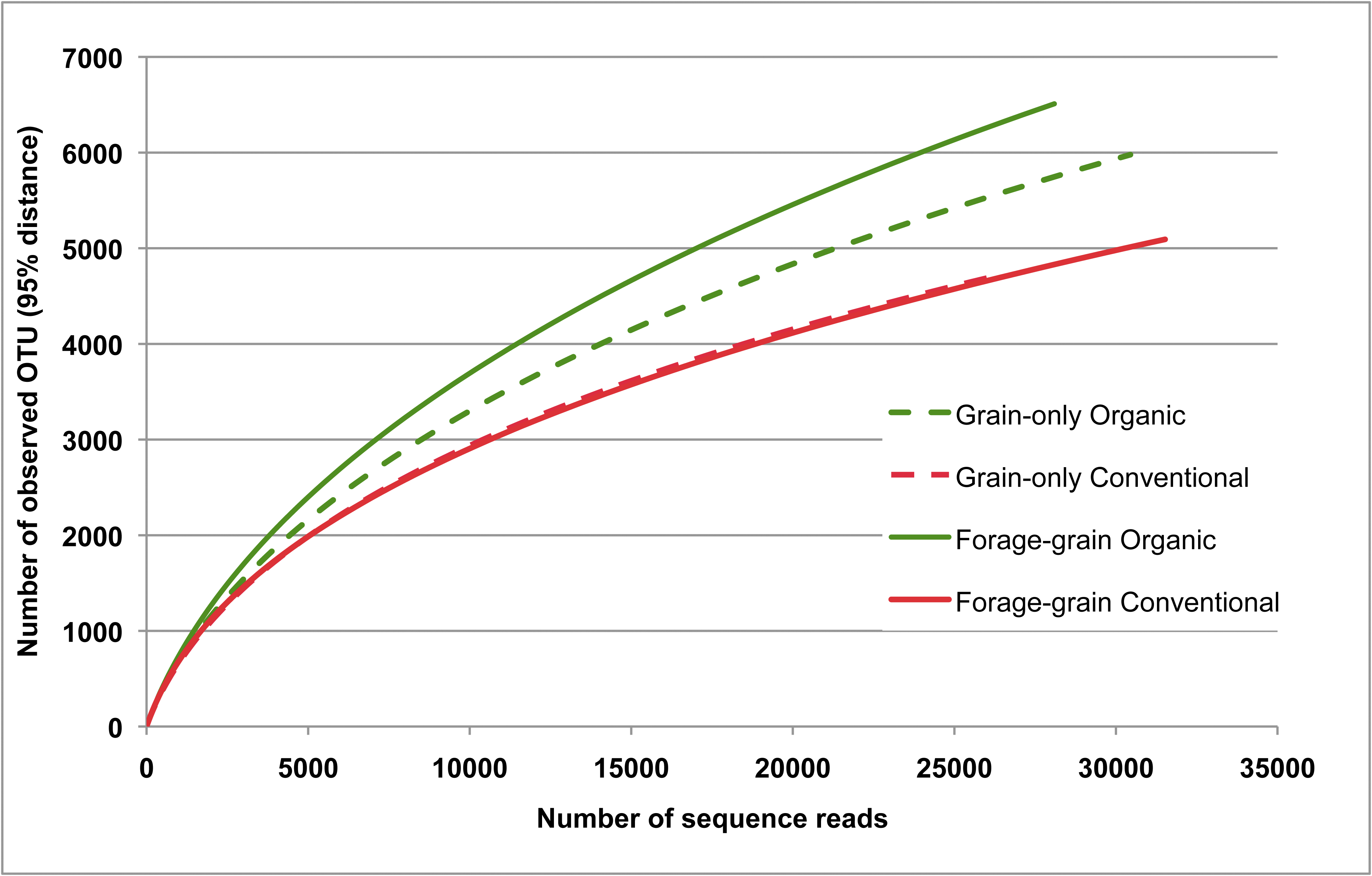

Supplement: Figure S1 — Rarefaction curves for pooled samples within each treatment at OTU cutoff of 0.05 distance. (TIF) [file pone.0051897.s001.tif]
