# Supplementary material for: Pyrosequencing Reveals the Influence of Organic and Conventional Farming Systems on Bacterial Communities
Source: PLoS One. 2012 Dec 19;7(12):e51897. doi: 10.1371/journal.pone.0051897 (PMC3526490; doi:10.1371/journal.pone.0051897)
Supplement: Table S1 — Phylogenetic composition of putative bacterial genera in Actinobacteria phylum determined using 16S rRNA pyrosequencing (DOC) [file pone.0051897.s003.doc]

| **Table S1.** Phylogenetic composition of putative bacterial genera in *Actinobacteria* phylum determined using 16S rRNA pyrosequencing | | | | | | | | | | | | | |
| --- | --- | --- | --- | --- | --- | --- | --- | --- | --- | --- | --- | --- | --- |
| Family; Genus | Rotation (Grain-Only) | | Rotation (Forage-Grain) | | | | | SEM | *P*-value | | | | |
|  | Management | | | | | | |  | Rotation | | Management | | Rotation × Management |
|  | Organic | Conventional | | Organic | | Conventional | |  |  |  |  |  |  |
|  | -------------------------------------------- Phylum, ***Actinobacteria*** ------------------------------------------------ | | | | | | | | | | | | |
| *Mycobacteriaceae*; *Mycobacterium* | 0.3 | 0.5 | 0.2 | | 0.2 | | 0.26 | | | 0.48 | 0.76 | 0.91 | |
| *Geodermatophilaceae*; *Blastococcus* | 0.8^b^ | 1.7^a^ | 1.2^a,b^ | | 1.7^a^ | | 0.19 | | | 0.27 | 0.002 | 0.23 | |
| *Kineosporiaceae*; *Kineosporia* | 0.5 | 0.3 | 0.5 | | 0.2 | | 0.25 | | | 0.61 | 0.31 | 0.72 | |
| *Intrasporangiaceae*; *Lapillicoccus* | 0.3^b^ | 0.4^a,b^ | 0.2^b^ | | 0.6^a^ | | 0.09 | | | 0.36 | 0.01 | 0.11 | |
| *Microbacteriaceae*; *Microbacterium* | 1.2 | 0.4 | 0.3 | | 0.1 | | 0.30 | | | 0.16 | 0.26 | 0.91 | |
| *Micrococcaceae*; *Arthrobacter* | 2.6 | 4.6 | 2.5 | | 3.6 | | 0.97 | | | 0.59 | 0.10 | 0.76 | |
| *Micromonosporaceae*; *Actinoplanes* | 0.8 | 0.7 | 1.1 | | 0.6 | | 0.38 | | | 0.90 | 0.47 | 0.54 | |
| *Micromonosporacea*; unclassified | 1.2 | 1.4 | 1.5 | | 1.8 | | 0.18 | | | 0.08 | 0.17 | 0.67 | |
| *Nocardioidaceae*; *Marmoricola* | 0.8 | 0.8 | 0.6 | | 0.4 | | 0.35 | | | 0.44 | 0.81 | 0.81 | |
| *Nocardioidaceae*; *Nocardioides* | 1.1 | 1.3 | 0.6 | | 0.7 | | 0.41 | | | 0.24 | 0.71 | 0.92 | |
| *Propionibacteriaceae*; *Microlunatus* | 1.1^a.b^ | 2.0^a^ | 0.8^b^ | | 2.1^a^ | | 0.31 | | | 0.78 | 0.004 | 0.66 | |
| *Pseudonocardiaceae*; *Pseudonocardia* | 0.9^b^ | 1.7^a,b^ | 1.3^b^ | | 2.6^a^ | | 0.24 | | | 0.02 | 0.001 | 0.31 | |
| *Pseudonocardiaceae*; unclassified | 0.3 | 0.4 | 0.3 | | 0.5 | | 0.07 | | | 0.34 | 0.05 | 0.23 | |
| *Solirubrobacteriaceae*; *Solirubrobacter* | 0.6^b^ | 1.7^a^ | 0.8^b^ | | 1.6^a^ | | 0.29 | | | 0.88 | 0.003 | 0.48 | |
| *Streptomycetaceae*; *Streptomyces* | 0.1 | 0.6 | 0.3 | | 0.5 | | 0.33 | | | 0.34 | 0.92 | 0.42 | |
| *Rubrobacteriaceae*; *Rubrobacter* | 0.5^b^ | 1.0^a^ | 0.3^b^ | | 1.4^a^ | | 0.24 | | | 0.57 | 0.005 | 0.30 | |
| Unclassified Actinobacteria | 11.9^a,b^ | 15.6^a,b^ | 9.9^b^ | | 17.4^a^ | | 1.81 | | | 0.97 | 0.01 | 0.33 | |
| ^a,b,c^ Means for main effects (rotation or management) are significantly different at *P* < 0.05.  ^A, B, C^ Means for the interaction between rotation and system are significantly different at *P* < 0.05. | | | | | | | | | | | | | |
