# Supplementary material for: Pyrosequencing Reveals the Influence of Organic and Conventional Farming Systems on Bacterial Communities
Source: PLoS One. 2012 Dec 19;7(12):e51897. doi: 10.1371/journal.pone.0051897 (PMC3526490; doi:10.1371/journal.pone.0051897)
Supplement: Table S2 — Phylogenetic composition of putative bacterial genera in Proteobacteria phylum determined using 16S rRNA pyrosequencing (DOC) [file pone.0051897.s004.doc]

| **Table S2.** Phylogenetic composition of putative bacterial genera in *Proteobacteria* phylum determined using 16S rRNA pyrosequencing | | | | | | | | | | | | |
| --- | --- | --- | --- | --- | --- | --- | --- | --- | --- | --- | --- | --- |
| Class; Family; Genus | Rotation (Grain-Only) | | | Rotation (Forage-Grain) | | | | SEM | *P*-value | | | |
|  | Management | | | Management | | | |  | Rotation | | Management | Rotation × Management |
|  | Organic | | Conventional | Organic | | Conventional | |  |  |  |  |  |
|  | ------------------------------------------------Phylum, ***Proteobacteria*** --------------------------------------------------- | | | | | | | | | | | |
| ***Alphaproteobacteria*** | 20.5 | 15.9 | | 18.2 | 15.0 | | 2.39 | | | 0.55 | 0.15 | 0.85 |
| *Caulobacteraceae*; *Brevundimonas* | 1.7^a^ | 0.1^b^ | | 0.2^b^ | 0.01^b^ | | 0.11 | | | 0.15 | 0.03 | 0.78 |
| *Caulobacteraceae*; *Phenylobacterium* | 0.5 | 0.3 | | 0.7 | 0.3 | | 0.28 | | | 0.83 | 0.42 | 0.80 |
| *Bradyrhizobiaceae*; *Balneimonas* | 0.4 | 0.6 | | 0.6 | 0.6 | | 0.31 | | | 0.74 | 0.72 | 0.68 |
| *Bradyrhizobiaceae*; *Bradyrhizobium* | 0.5 | 0.8 | | 0.9 | 0.9 | | 0.16 | | | 0.13 | 0.41 | 0.29 |
| *Bradyrhizobiaceae*; unclassified | 0.7 | 0.6 | | 0.9 | 0.7 | | 0.28 | | | 0.76 | 0.62 | 1.00 |
| *Hyphomicrobiaceae*; *Devosia* | 0.6 | 0.4 | | 0.6 | 0.9 | | 0.27 | | | 0.36 | 0.24 | 0.41 |
| *Shinella_genera_incertae_sedis*; unclassified | 0.6 | 0.4 | | 0.3 | 0.1 | | 0.23 | | | 0.21 | 0.42 | 0.64 |
| *Xanthobacteraceae*; uncultured | 0.5^b^ | 0.8^a,b^ | | 0.7^a,b^ | 1.1^a^ | | 0.15 | | | 0.10 | 0.05 | 0.64 |
| *Acetobacteraceae*; unclassified | 0.7 | 0.7 | | 1.0 | 0.8 | | 0.39 | | | 0.70 | 0.90 | 0.76 |
| DA111; unclassified | 0.2 | 0.6 | | 0.4 | 0.7 | | 0.14 | | | 0.61 | 0.23 | 0.72 |
| *Rhodospirillaceae*; *Skermanella* | 0.8^b^ | 1.5^a,b^ | | 1.3^ab^ | 2.6^a^ | | 0.30 | | | 0.02 | 0.005 | 0.36 |
| Wr0007; unclassified | 0.4 | 0.5 | | 1.2 | 0.8 | | 0.37 | | | 0.22 | 0.83 | 0.55 |
| *Erythrobacteraceae*; *Altererythrobacter* | 0.5 | 0.3 | | 0.2 | 0.1 | | 0.22 | | | 0.31 | 0.65 | 0.99 |
| ***Betaproteobacteria*** | 10.3 | 6.3 | | 8.7 | 5.1 | | 2.00 | | | 0.42 | 0.04 | 0.93 |
| *Alcaligenaceae*; *Achromobacter* | 0.6 | 0.1 | | 0.0 | 0.1 | | 0.19 | | | 0.39 | 0.88 | 0.47 |
| *Burkholderiaceae*; *Burkholderia* | 0.6 | 0.0 | | 0.1 | 0.0 | | 0.19 | | | 0.29 | 0.14 | 0.99 |
| *Comamonadaceae*; *Methylibium* | 1.0 | 0.9 | | 1.6 | 0.9 | | 0.47 | | | 0.54 | 0.44 | 0.62 |
| *Comamonadaceae;* *Variovorax* | 1.0 | 0.4 | | 0.8 | 0.4 | | 0.34 | | | 0.70 | 0.19 | 0.94 |
| *Oxalobacteraceae*; *Duganella* | 0.6 | 0.7 | | 0.3 | 0.3 | | 0.28 | | | 0.31 | 0.99 | 0.74 |
| *Oxalobacteraceae*; *Massilia* | 0.8 | 0.4 | | 0.3 | 0.1 | | 0.27 | | | 0.17 | 0.25 | 0.72 |
| *Oxalobacteraceae*; unclassified | 0.4 | 0.7 | | 0.9 | 0.5 | | 0.18 | | | 0.46 | 0.93 | 0.08 |
| *Nitrosomonadaceae*; uncultured | 0.2 | 0.2 | | 0.6 | 0.2 | | 0.10 | | | 0.11 | 0.17 | 0.16 |
| ***Gammaproteobacteria*** | 11.4 | 7.1 | | 7.7 | 3.6 | | 2.72 | | | 0.13 | 0.08 | 0.67 |
| *Enterobacteriaceae*; *Pantoea* | 0.5 | 0.2 | | 0.0 | 0.0 | | 0.17 | | | 0.20 | 0.73 | 0.98 |
| *Pseudomonadaceae*; *Pseudomonas* | 4.3 | 3.9 | | 1.7 | 0.5 | | 2.02 | | | 0.10 | 0.52 | 0.44 |
| *Sinobacteraceae*; unclassified | 1.4 | 0.9 | | 1.0 | 1.1 | | 0.47 | | | 0.85 | 0.69 | 0.61 |
| *Xanthomonadaceae*; *Arenimonas* | 1.5 | 0.4 | | 1.0 | 0.6 | | 0.41 | | | 0.94 | 0.12 | 0.47 |
| *Xanthomonadaceae*; *Pseudoxanthomonas* | 0.1 | 0.0 | | 0.6 | 0.0 | | 0.18 | | | 0.56 | 0.24 | 0.62 |
| *Xanthomonadaceae*; *Stenotrophomonas* | 0.7^a^ | 0.0^b^ | | 0.3^a^ | 0.0^b^ | | 0.25 | | | 0.67 | 0.04 | 0.71 |
| *Xanthomonadaceae*; *Thermomonas* | 0.6 | 0.2 | | 0.2 | 0.1 | | 0.21 | | | 0.44 | 0.41 | 0.75 |
| *Xanthomonadaceae*; *Xanthomonas* | 0.5 | 0.1 | | 0.2 | 0.0 | | 0.18 | | | 0.47 | 0.33 | 0.87 |
| *Xanthomonadaceae*; unclassified | 0.7 | 0.5 | | 1.1 | 0.5 | | 0.36 | | | 0.69 | 0.18 | 0.45 |
| ***Deltaproteobacteria*** | 1.0^b^ | 2.7^a,b^ | | 3.4^a^ | 3.5^a^ | | 0.05 | | | 0.009 | 0.10 | 0.13 |
| Unclassified Proteobacteria | 3.9 | 4.5 | | 6.1 | 4.4 | | 0.58 | | | 0.03 | 0.26 | 0.03 |
| ^a,b,c^ Means for main effects (rotation or management) are significantly different at *P* < 0.05.  ^A, B, C^ Means for the interaction between rotation and system are significantly different at *P* < 0.05. | | | | | | | | | | | | |
