# Supplementary material for: Pyrosequencing Reveals the Influence of Organic and Conventional Farming Systems on Bacterial Communities
Source: PLoS One. 2012 Dec 19;7(12):e51897. doi: 10.1371/journal.pone.0051897 (PMC3526490; doi:10.1371/journal.pone.0051897)
Supplement: Table S3 — Phylogenetic composition of putative bacterial genera in Acidobacteria, Bacteroidetes, Chloroflexi, Firmicutes, Gemmatimonadetes, and Planctomycetes phyla determined using 16S rRNA pyrosequencing (DOC) [file pone.0051897.s005.doc]

| **Table S3.** Phylogenetic composition of putative bacterial genera in *Acidobacteria, Bacteroidetes, Chloroflexi, Firmicutes, Gemmatimonadetes*, and *Planctomycetes* phyla determined using 16S rRNA pyrosequencing | | | | | | | | | | | | | | |
| --- | --- | --- | --- | --- | --- | --- | --- | --- | --- | --- | --- | --- | --- | --- |
| Family; Genus | Rotation (Grain-Only) | | | | Rotation (Forage-Grain) | | | | | SEM | *P*-value | | | |
|  | Management | | | | | | | | |  | Rotation | | Management | Rotation × Management |
|  | Organic | Conventional | | | Organic | | Conventional | | |  |  |  |  |  |
|  | ------------------------------------------------Phylum, ***Acidobacteria*** ----------------------------------------------------- | | | | | | | | | | | | | |
| *Acidobacteriaceae*; *Chloroacidobacterium* | 0.4 | 0.6 | | | 0.9 | 0.5 | | | | 0.33 | | 0.51 | 0.97 | 0.41 |
| *Acidobacteriaceae*; uncultured | 7.1 | 8.6 | | | 8.3 | 11.0 | | | | 0.26 | | 0.46 | 0.40 | 0.86 |
| *Acidobacteria*; unclassified | 1.1 | 1.7 | | | 4.2 | 2.2 | | | | 0.85 | | 0.06 | 0.76 | 0.18 |
|  | ------------------------------------------------Phylum, ***Bacteroidetes*** ----------------------------------------------------- | | | | | | | | | | | | | |
| *Flavobacteriaceae*; *Flavobacterium* | 0.5 | | 0.8 | | 1.5 | 0.4 | | | 0.45 | | | 0.77 | 0.38 | 0.12 |
| *Chitinophagaceae*; unclassified | 0.6 | | 0.7 | | 1.0 | 0.5 | | | 0.36 | | | 0.97 | 0.52 | 0.38 |
| *Cytophagaceae*; *Hymenobacter* | 0.6 | | 0.1 | | 0.1 | 0.0 | | | 0.18 | | | 0.19 | 0.41 | 0.76 |
|  | -----------------------------------------------Phylum, ***Chloroflexi*** ------------------------------------------------------- | | | | | | | | | | | | | |
| *Anaerolineaceae*; unclassified | 0.6 | 1.2 | | 0.9 | | 1.2 | | 0.41 | | | | 0.63 | 0.25 | 0.71 |
| *Chloroflexaceae*; *Roseiflexus* | 0.6 | 1.5 | | 1.1 | | 2.3 | | 0.28 | | | | 0.02 | 0.002 | 0.52 |
| Unclassified | 0.4 | 0.6 | | 0.7 | | 0.5 | | 0.32 | | | | 0.76 | 0.97 | 0.52 |
|  | ------------------------------------------------Phylum, ***Firmicutes*** --------------------------------------------------------- | | | | | | | | | | | | | |
| *Bacillaceae*; *Bacillus* | 0.4 | 0.5 | | 0.2 | | 0.4 | | 0.25 | | | | 0.42 | 0.45 | 0.66 |
|  | ------------------------------------------------Phylum, ***Gemmatimonadetes*** ---------------------------------------------- | | | | | | | | | | | | | |
| *Gemmatimonadaceae*; *Gemmatimonas* | 1.9 | 1.1 | | 2.7 | | 1.8 | | 0.73 | | | | 0.29 | 0.23 | 0.87 |
| *Gemmatimonadaceae*; unclassified | 1.8 | 2.7 | | 4.1 | | 3.9 | | 1.16 | | | | 0.11 | 0.63 | 0.53 |
| Unclassified | 0.1 | 0.2 | | 0.4 | | 0.5 | | 0.23 | | | | 0.27 | 0.50 | 0.78 |
|  | -----------------------------------------------Phylum, ***Planctomycetes*** --------------------------------------------------- | | | | | | | | | | | | | |
| Unclassified | 0.9 | 0.9 | | 1.3 | | 0.9 | | 0.27 | | | | 0.59 | 0.51 | 0.48 |
